# Supplementary material for: Effect of Parylene C on the Corrosion Resistance of Bioresorbable Cardiovascular Stents Made of Magnesium Alloy ‘Original ZM10’
Source: Materials (Basel). 2022 Apr 26;15(9):3132. doi: 10.3390/ma15093132 (PMC9102321; doi:10.3390/ma15093132)
Supplement: Supplementary file 1 [file materials-15-03132-s001.zip › materials-1666745-supplementary.pdf]

Supporting Information

Effect of Parylene C on the Corrosion Resistance of Bioresorbable Cardiovascular Stents Made of Magnesium Alloy 'Original ZM10'

Makoto Sasaki [1,*], Wei Xu [1,*], Yuki Koga [2], Yuki Okazawa [3], Akira Wada [2], Ichiro Shimizu [4] and Takuro Niidome [1]

[1] Faculty of Advanced Science and Technology, Kumamoto University, Kumamoto 860-8555, Japan; niidome@kumamoto-u.ac.jp
[2] Japan Medical Device Technology, Co., Ltd., Kumamoto 861-2202, Japan; a.wada@jmdt.co.jp (A.W.); y.koga@jmdt.co.jp (Y.K.)
[3] Fuji Light Metal, Co., Ltd., Kumamoto 869-0192, Japan; yuuki-okazawa@fujisash.net
[4] Department of Mechanical Engineering, Okayama University of Science, Okayama 700-0005, Japan; shimizu@mech.ous.ac.jp
[*] Correspondence: sasakimakoto@kumamoto-u.ac.jp (M.S.); xuwei@kumamoto-u.ac.jp (W.X.); Tel.: +81-96-342-3288 (M.S.); +81-96-342-3668 (W.X.)

Table S1. Elemental composition (wt.%) of Original ZM10.

Alloying Elements [wt.%]	Mg	Al	Zn	Zr	Mn	Rare Earth			
						Y	Dy	Nd	Gd
Original ZM10	Balance	–	1.45	0.14	≤0.20	–	–	–	–

Table S2. Specifications of the stent used in this study.

Material	Original ZM10
Strut width (μm)	116~150
Strut thickness (μm)	110
Crossing profile (mm)	1.2–1.3
Drug dose	1.0 μg/mm^2 (Sirolimus)

Table S3. Mg ion release (%) from Mg alloy stents after balloon expansion.

Mg ion release [%]	bare	HF	HF/parylene C	HF/polymer	HF/parylene C/polymer
1 month	38.1±4.9	12.8±0.9	0.1±0.01	8.7±1.0	0.2±0.2
2 months	–	38.1±9.7	0.1±0.01	–	–
3 months	–	60.6±7.7	0.2±0.01	–	–
4 months	–	74.1±6.4	0.2±0.01	–	–
5 months	–	77.4±2.4	0.2±0.01	–	–
6 months	–	78.3±0.1	0.2±0.01	–	–

The data represent the mean value for n = 5 with the standard deviations of the means.

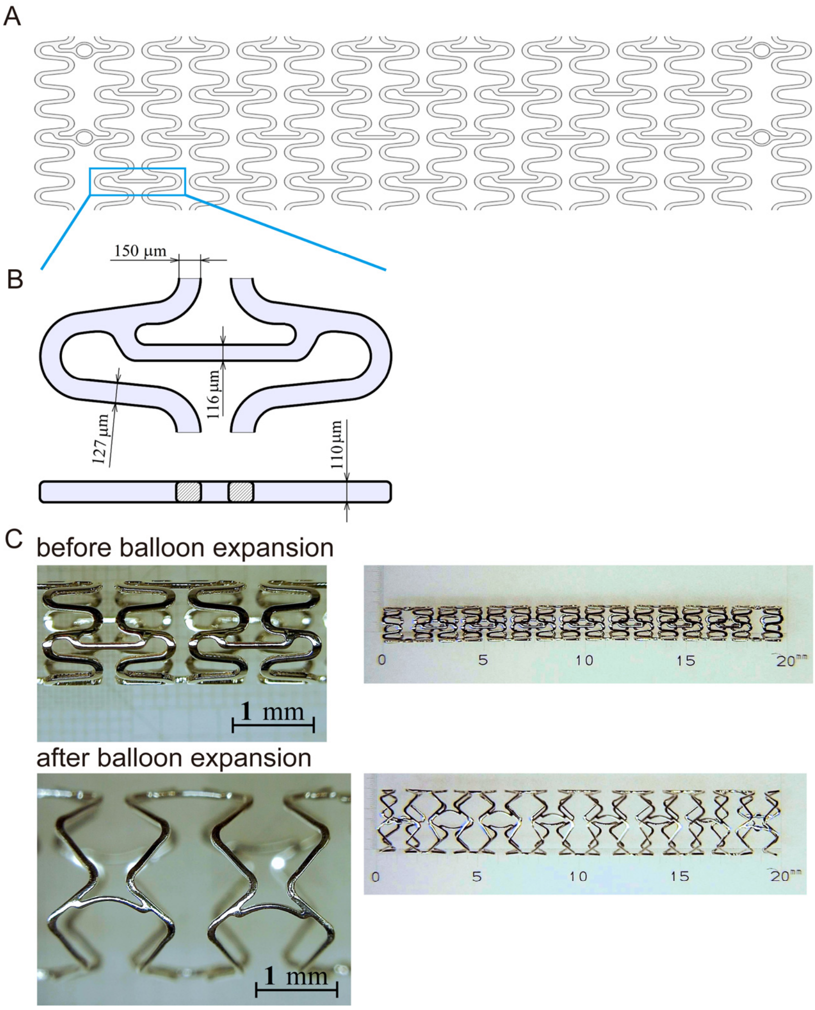

Figure S1. Structure of the Mg alloy stent, 3.0 × 20 mm with six-crown two-link design and square-shaped struts. (**A**) Developed structure of the stent. (**B**) Structure of one unit of the stent. (**C**) Microscopy images of the stent before (upper) and after (lower) balloon expansion.
